# Higher hemoglobin levels using darbepoetin alfa and kidney outcomes in advanced chronic kidney disease without diabetes: a prespecified secondary analysis of the PREDICT trial

**DOI:** 10.1007/s10157-023-02362-w

**Published:** 2023-06-08

**Authors:** Shoichi Maruyama, Shimon Kurasawa, Terumasa Hayashi, Masaomi Nangaku, Ichiei Narita, Hideki Hirakata, Kenichiro Tanabe, Satoshi Morita, Yoshiharu Tsubakihara, Enyu Imai, Tadao Akizawa, PREDICT Investigators, Takeyuki Hiramatsu, Hirofumi Tamai, Yoshiyasu Iida, Tomohiro Naruse, Hideto Oishi, Shunya Uchida, Hideaki Shimizu, Kunio Morozumi, Hisashi Kurata, Nobuhito Hirawa, Saori Nishio, Yukio Yuzawa, Makoto Mizutani, Isao Aoyama, Hideaki Yoshida, Kouji Kaneda, Satoshi Suzuki, Hiroki Adachi, Eriko Kinugasa, Kei Kurata, Hiroshi Morinaga, Yusuke Tsukamoto, Kazuhiro Tsuruya, Ryoichi Ando, Shizunori Ichida, Teiichi Tamura, Takao Masaki, Takashi Wada, Hirokazu Honda, Junichiro Yamamoto, Yoshitaka Isaka, Eri Muso, Yasuhiro Komatsu, Norimi Ohashi, Taiga Hara, Kiyoshi Ikeda, Kazuyoshi Okada, Tetsuhiko Yoshida, Seiya Okuda, Hiromichi Suzuki, Takeshi Nakanishi, Harumichi Higashi, Arimasa Shirasaki, Shuichiro Endo, Yutaka Osawa, Ryuji Aoyagi, Yasuhiko Tomino, Tetsu Akimoto, Tsuyoshi Watanabe, Jiro Toyonaga, Motoko Tanaka, Yoshitaka Ishibashi, Shigehiro Uezono, Masako Sakakibara, Hajime Yamazaki, Hideki Takano, Hirofumi Ikeda, Takuma Takata, Hiroshi Yamashita, Kunihiro Yamagata, Toshinobu Sato, Ashio Yoshimura, Keiichi Tamagaki, Kazuhiro Sonomura, Akira Iguchi, Masahito Tamura, Ryota Yasukawa, Manei Oku

**Affiliations:** 1 Department of Nephrology, Nagoya University Graduate School of Medicine, 65 Tsurumai-Cho, Showa-Ku, Nagoya, 466-8550 Japan; 2 Department of Clinical Research Education, Nagoya University Graduate School of Medicine, Nagoya, Japan; 3 Department of Kidney Disease and Hypertension, Osaka General Medical Center, Osaka, Japan; 4 Division of Nephrology and Endocrinology, The University of Tokyo, Tokyo, Japan; 5 Division of Clinical Nephrology and Rheumatology, Niigata University, Niigata, Japan; 6 Fukuoka Renal Clinic, Fukuoka, Japan; 7 Division of Health Data Science, Translational Research Center for Medical Innovation, Kobe, Japan; 8 Pathophysiology and Bioregulation, St. Marianna University Graduate School of Medicine, Kanagawa, Japan; 9 Biomedical Statistics and Bioinformatics, Kyoto University, Kyoto, Japan; 10 Graduate School of Health Care Sciences, Jikei Institute, Osaka, Japan; 11 Nakayamadera Imai Clinic, Takarazuka, Japan; 12 Division of Nephrology, Showa University School of Medicine, Tokyo, Japan

**Keywords:** Renal anemia, CKD, Kidney, eGFR slope, Proteinuria

## Abstract

**Background:**

In the primary analysis of the PREDICT trial, a higher hemoglobin target (11–13 g/dl) with darbepoetin alfa did not improve renal outcomes compared with a lower hemoglobin target (9–11 g/dl) in advanced chronic kidney disease (CKD) without diabetes. Prespecified secondary analyses were performed to further study the effects of targeting higher hemoglobin levels on renal outcomes.

**Methods:**

Patients with an estimated glomerular filtration rate (eGFR) 8–20 ml/min/1.73 m^2^ without diabetes were randomly assigned 1:1 to the high- and low-hemoglobin groups. The differences between the groups were evaluated for the following endpoints and cohort sets: eGFR and proteinuria slopes, assessed using a mixed-effects model in the full analysis set and the per-protocol set that excluded patients with off-target hemoglobin levels; the primary endpoint of composite renal outcome, evaluated in the per-protocol set using the Cox model.

**Results:**

In the full analysis set (high hemoglobin, n = 239; low hemoglobin, n = 240), eGFR and proteinuria slopes were not significantly different between the groups. In the per-protocol set (high hemoglobin, n = 136; low hemoglobin, n = 171), the high-hemoglobin group was associated with reduced composite renal outcome (adjusted hazard ratio: 0.64; 95% confidence interval: 0.43–0.96) and an improved eGFR slope (coefficient: + 1.00 ml/min/1.73 m^2^/year; 95% confidence interval: 0.38–1.63), while the proteinuria slope did not differ between the groups.

**Conclusions:**

In the per-protocol set, the high-hemoglobin group demonstrated better kidney outcomes than the low-hemoglobin group, suggesting a potential benefit of maintaining higher hemoglobin levels in patients with advanced CKD without diabetes.

**Clinical trial registration:**

Clinicaltrials.gov (identifier: NCT01581073).

**Supplementary Information:**

The online version contains supplementary material available at 10.1007/s10157-023-02362-w.

## Introduction

Chronic kidney disease (CKD) is a globally significant burden that affects society, with an estimated prevalence of 10–13% worldwide. Moreover, the number of patients with CKD requiring renal replacement therapy is estimated to be 4.9–7.0 million [1, 2]. Anemia is a common complication among patients with CKD not requiring dialysis [3, 4]. The prevalence of anemia increases as the stage of CKD progresses, from 8% at stage 1 to 53% at stage 5 [5]. Observational studies have suggested that anemia may be a biomarker independently associated with increased cardiovascular (CV) and kidney events [6–8]. Erythropoiesis-stimulating agents (ESAs) have been widely used to treat renal anemia in patients with CKD on dialysis and those not requiring dialysis. However, interventional studies using ESAs in patients with CKD not requiring dialysis have reported conflicting results [9–14], and the optimal target hemoglobin levels for patients with CKD are unknown [10–13].

A small randomized controlled trial (RCT) conducted by Gouva et al. has demonstrated favorable effects of early intervention using erythropoietin alfa on renal outcomes [9]. Thereafter, three large RCTs failed to show the clinical benefits of targeting higher hemoglobin levels. In the Cardiovascular Risk Reduction by Early Anemia Treatment with Epoetin Beta (CREATE) trial, using epoetin beta to target high hemoglobin levels (13.0–15.0 g/dl) vs. low hemoglobin levels (10.5–11.5 g/dl) also failed to reduce the incidence of CV events, while the number of patients requiring dialysis therapy significantly increased [11]. In the Correction of Hemoglobin and Outcomes in Renal Insufficiency (CHOIR) trial, patients with CKD not requiring dialysis were randomly assigned to target either a high (13.0–13.5 g/dl) or a low hemoglobin level (10.5–11.0 g/dl) using epoetin alfa [12]. However, targeting higher hemoglobin levels was associated with a significantly higher risk of a composite outcome of death and CV events. In the Trial to Reduce Cardiovascular Events with Aranesp Therapy (TREAT), patients with CKD not requiring dialysis who have diabetes were randomly assigned to a placebo and a group receiving darbepoetin alfa to achieve a hemoglobin level of ≥ 13 g/dl [13]. Similarly, darbepoetin alfa failed to reduce the risk of the two primary composite outcomes (CV outcome [death or a CV event] and renal outcome [death or a renal event]). However, it significantly increased the risk of stroke. A meta-analysis report has concluded that targeting higher hemoglobin levels with ESAs reduces the need for blood transfusions but increases the risk of CV and kidney events in patients with CKD [16, 17].

Recently, hypoxia-inducible factor prolyl-hydroxylase inhibitors (HIF-PHIs) have been available. Almost all the studies have successfully demonstrated that the efficiency of HIF-PHIs in treating anemia is comparable with that of ESAs [18–21]. However, no clinical studies have ever shown the clear advantage of HIF-PHIs over ESAs or placebo on the kidney or CV outcomes in patients with CKD not requiring dialysis. Moreover, the optimal target hemoglobin levels using HIF-PHIs for patients with CKD not requiring dialysis have not been previously reported [22].

Tsubakihara et al. conducted an RCT in Japan to investigate the renal protective effects [14, 15]. Although the primary analysis was negative, post-hoc analyses demonstrated that maintaining hemoglobin levels (11–13 g/dl) with darbepoetin alfa improved renal outcomes as compared to maintaining hemoglobin levels (9–11 g/dl) with epoetin alfa in patients at stage 5 CKD not requiring dialysis, particularly those without diabetes. Based on these findings, we conducted an RCT, namely ‘Prevention of end-stage kidney disease (ESKD) by Darbepoetin Alfa in CKD Patients with Non-diabetic Kidney Disease (PREDICT) trial.’ This trial aimed to prove our hypothesis that targeting a higher hemoglobin level (11–13 g/dl) with darbepoetin alfa would prevent ESKD as compared to targeting a lower hemoglobin level (9–11 g/dl) in patients with advanced CKD without diabetes [23]. The primary analysis revealed that targeting a higher hemoglobin level did not significantly improve renal outcomes compared with targeting a lower hemoglobin level [24]. It is noteworthy that in the PREDICT trial, the prognosis of the high-hemoglobin group demonstrated a tendency to improve (hazard ratio [HR] 0.78; 95% confidence interval [CI] 0.60–1.03), whereas the prognosis in the three studies mentioned above [11–13] yielded opposite results.

In this prespecified secondary analysis of the PREDICT trial, we aimed to further clarify the effects of targeting hemoglobin levels using darbepoetin alfa on renal outcomes, including the primary endpoint of composite renal endpoint in the per-protocol set (PPS) and the secondary endpoints of eGFR and proteinuria slopes in the full analysis set (FAS) and PPS, in patients with advanced CKD without diabetes.

## Materials and methods

### Study design

The PREDICT trial was a multicenter, randomized, open-label, parallel-group study performed in patients with an estimated glomerular filtration rate (eGFR) of 8–20 ml/min/1.73 m^2^, renal anemia with a hemoglobin level < 10 g/dl and no diabetes. The detailed study design and methods have been previously described [23, 24]. The trial was designed, implemented, and overseen by the PREDICT Executive Committee, along with representatives of the Translational Research Center for Medical Innovation, Kobe, Japan, a third-party organization. The study was registered at Clinicaltrials.gov (identifier: NCT01581073).

### Study population and randomization

A total of 491 patients with CKD without diabetes, aged 20–85 years, with an eGFR of 8–20 ml/min/1.73 m^2^ calculated with the Japanese equation [25] and with a hemoglobin level < 10 g/dl, were randomly assigned to a high- (11–13 g/dl) or a low-hemoglobin (9–11 g/dl) group using darbepoetin alfa in a 1:1 ratio. The registration period was from December 2011 to June 2014, and the observation period lasted two years after the last patient enrollment, up to June 2016.

**Fig. 1:**
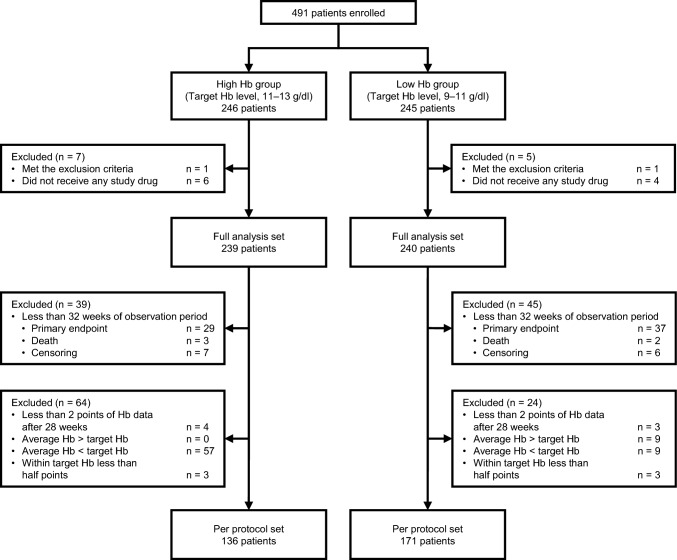
Diagram of patient flow. *Hb* hemoglobin

PPS was defined as patients in the FAS whose hemoglobin level had been measured at least twice after 28 weeks (inevitably, the observation period was ≥ 32 weeks), the mean of which was within the target range, and more than half of each measurement was within the target range (Fig. 1).

### Outcomes

The outcomes of the PREDICT trial have also been described previously [23, 24]. The primary endpoint was the onset of a composite renal endpoint, including the initiation of dialysis, kidney transplantation, reaching an eGFR of 6 ml/min/1.73 m^2^, and ≥ 50% of reduction in eGFR. Among the prespecified secondary endpoint, the initiation of dialysis, ≥ 50% reduction in eGFR, all-cause death, composite CV event, stroke, myocardial infarction, and the development of malignancy have already been reported in our previous report [24]. Here, in addition to the composite renal endpoint in the PPS, the unreported and prespecified secondary endpoints of changes in eGFR and proteinuria in the FAS and PPS were assessed. Serious adverse events including all-cause death, CV events, Malignant neoplasms in the PPS were also assessed.

### Statistical analysis

The methods of statistical analysis were described previously [23, 24]. The differences in the primary endpoint between the study groups in the PPS were assessed using the Kaplan–Meier method with a log-rank test. Multivariable Cox regression analysis was also performed to estimate HRs with 95% CIs for the composite renal endpoint in the PPS. The analyses were censored on the date of death and the end of the observation period (2 years from baseline) without a composite renal endpoint. The proportional hazards assumption was checked using Schoenfeld residual, and no violation was recorded. Changes in eGFR and proteinuria were assessed as eGFR slope (ml/min/1.73 m^2^/year) and proteinuria slope (g/gCr/year), respectively. The eGFR and proteinuria slopes were calculated using a linear mixed effects model with an unstructured variance–covariance matrix and patient-level random slopes and intercepts. The effects of the higher hemoglobin targeting on eGFR and proteinuria slopes were evaluated by adding an interaction term between the high-hemoglobin group and time in the mixed-effects model using the FAS and PPS, respectively. The multivariable models were adjusted for sex, age, baseline eGFR, baseline hemoglobin, systolic blood pressure, and proteinuria. These covariates as well as the interaction terms between each covariate and time were included in the model.

All test statistics used in the analyses were the results of two-sided tests with a significance level of 5%. Data are reported as number (percentage), mean ± standard deviation, median (interquartile range), or estimated value with 95% CI as appropriate. Normality was assessed by inspecting histograms. All statistical analyses were performed using SAS 9.4 (SAS Institute Inc., Cary, NC, USA) and Stata/MP 17.0 (StataCorp., College Station, TX, USA).

## Results

### Analysis of cohort sets

**Fig. 2:**
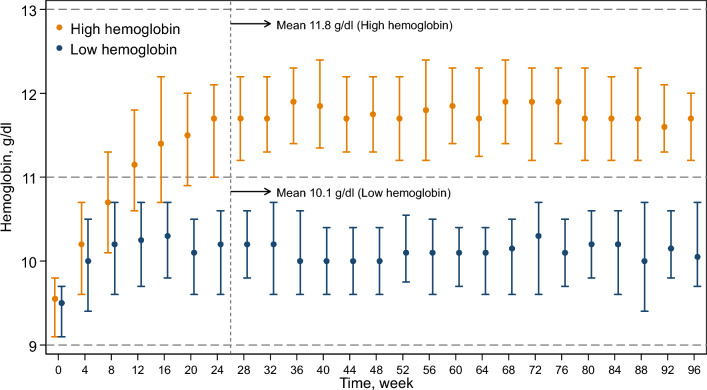
Transition of hemoglobin levels of the two groups in per-protocol set (n = 307). The plots with capped spikes are medians with interquartile ranges. Values are mean hemoglobin after 28 weeks in each study group. Target hemoglobin levels were 11–13 g/dl in the high-hemoglobin group and 9–11 g/dl in the low-hemoglobin group

Of the 479 patients in the FAS (high-hemoglobin group, 239; low-hemoglobin group, 240), 307 were included in the PPS (high-hemoglobin group, 136; low-hemoglobin group, 171) (Fig. 1). The patients’ characteristics at baseline are summarized in Table 1. There was no significant difference between the study groups. Figure 2 illustrates the transition of hemoglobin levels by study group in the PPS. The mean hemoglobin levels after 28 weeks were 11.8 g/dl and 10.1 g/dl in the high- and low-hemoglobin groups, respectively. The medians (interquartile ranges) darbepoetin alfa doses in each visit ranged from 67 to 100 µg/4 weeks and 33 to 55 µg/4 weeks in the high- and low-hemoglobin groups (Supplementary Fig. 1).

**Table 1:** Baseline characteristics in per-protocol set

	Total	High-hemoglobin group	Low-hemoglobin group
	n = 307	n = 136	n = 171
Male sex	171 (56)	73 (54)	98 (57)
Age, years	71 ± 11	71 ± 10	70 ± 11
Body mass index, kg/m^2^	22.2 ± 3.1	22.0 ± 3.2	22.4 ± 3.0
Smoking			
Current/ever	245 (80)	109 (80)	136 (80)
Never	34 (11)	13 (9.6)	21 (12)
Unknown	28 (9.1)	14 (10)	14 (8.2)
Etiology of chronic kidney disease			
Chronic glomerulonephritis	83 (27)	30 (22)	53 (31)
Hypertensive nephrosclerosis	164 (53)	79 (58)	85 (50)
Polycystic kidney disease	30 (10)	17 (12)	13 ( 8)
Others	30 (10)	10 ( 7)	20 (12)
History of cardiovascular disease			
Acute myocardial infarction	12 (3.9)	5 (3.7)	7 (4.1)
Heart failure	29 (9.4)	17 (12)	12 (7.0)
Stroke	27 (8.8)	13 (9.6)	14 (8.2)
Peripheral artery disease	5 (1.7)	1 (0.7)	4 (2.4)
Erythropoiesis-stimulating agents naive	214 (70)	97 (71)	117 (68)
Systolic blood pressure, mm Hg	132 ± 15	131 ± 17	132 ± 14
Diastolic blood pressure, mm Hg	72 ± 12	71 ± 12	73 ± 11
eGFR, ml/min/1.73 m^2^	14 ± 3	14 ± 3	14 ± 3
Chronic kidney disease			
Stage 4	122 (40)	59 (43)	63 (37)
Stage 5	185 (60)	77 (57)	108 (63)
Hemoglobin, g/dl	9.4 ± 0.5	9.4 ± 0.5	9.3 ± 0.6
Ferritin, ng/ml	148 (96–233)	144 (92–222)	150 (97–241)
Transferrin saturation, %	32 ± 11	32 ± 11	33 ± 11
Uric acid, mg/dl	7.2 ± 1.7	7.2 ± 1.8	7.2 ± 1.6
Albumin, g/dl	3.8 ± 0.4	3.8 ± 0.4	3.8 ± 0.4
Proteinuria, mg/dl	58 (21–116)	47 (20–113)	61 (25–117)
C-reactive protein, mg/dl	0.09 (0.03–0.20)	0.09 (0.03–0.20)	0.08 (0.03–0.20)
Iron supplements	91 (30)	42 (31)	49 (29)
Antihypertensive drugs	278 (91)	124 (91)	154 (90)
ACE inhibitors	38 (12)	17 (12)	21 (12)
Angiotensin II receptor blockers	187 (61)	91 (67)	96 (56)
Lipid-lowering drugs	116 (38)	55 (40)	61 (36)
Spherical carbonaceous adsorbent	63 (21)	27 (20)	36 (21)

*eGFR* estimated glomerular filtration rate, *ACE* angiotensin-converting enzyme

Continuous variables are presented as mean ± standard deviation if normally distributed, and as median (interquartile range) if non-normally distributed. Categorical variables are presented as n (percent)

### Changes in eGFR and proteinuria in FAS

The crude mean ± SD eGFR slopes in the high- and low-hemoglobin groups were − 3.27 ± 3.32 and − 3.77 ± 3.25 ml/min/1.73 m^2^/year, respectively. In the multivariable analysis, the high-hemoglobin group was not associated with eGFR slope (coefficient: + 0.59; 95% CI − 0.06 to + 1.24; *P* = 0.075), while male sex, lower age, and higher proteinuria at baseline were significantly associated with faster eGFR decline (Supplementary Fig. 2).

The crude mean ± SD proteinuria slopes in the high- and low-hemoglobin groups were + 0.65 ± 0.063 and + 0.62 ± 0.055 g/gCr/year, respectively, with no significant difference in the multivariable model (Supplementary Table 1).

### Composite renal endpoint in PPS

**Fig. 3:**
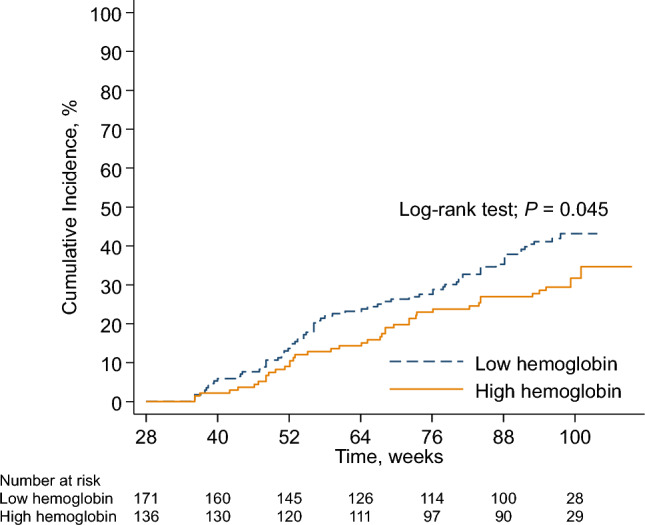
Cumulative incidence of the composite renal endpoint by study group in per-protocol set (n = 307). The composite renal endpoint includes initiation of maintenance dialysis, kidney transplantation, eGFR < 6 ml/min/1.73 m^2^, and a 50% reduction in eGFR. *eGFR* estimated glomerular filtration rate

**Fig. 4:**
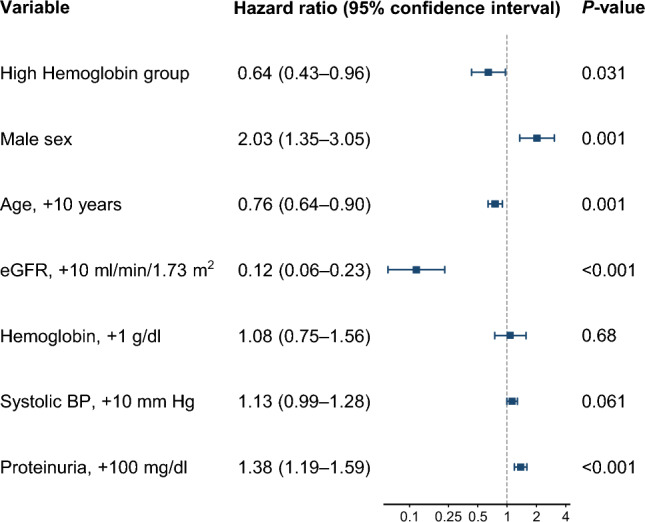
Multivariable Cox proportional hazard regression for the composite renal endpoint in per-protocol set (n = 307). The composite renal endpoint includes initiation of maintenance dialysis, kidney transplantation, eGFR < 6 ml/min/1.73 m^2^, and a 50% reduction in eGFR. *eGFR* estimated glomerular filtration rate, *BP* blood pressure

The composite renal endpoint occurred in 40 (29%) and 69 (40%) patients in the high- and low-hemoglobin groups, respectively. The Kaplan–Meier plots of the composite renal endpoint in the PPS are provided in Fig. 3 (log-rank test: *P* = 0.045). In the multivariable Cox proportional hazard regression analysis, high hemoglobin levels indicated a lower risk of composite renal endpoint (adjusted HR 0.64; 95% CI 0.43–0.96; *P* = 0.031). Male sex, lower age, lower eGFR, and higher proteinuria were associated with a higher risk of composite renal endpoint (Fig. 4).

### Changes in eGFR and proteinuria and adverse events in PPS

**Fig. 5:**
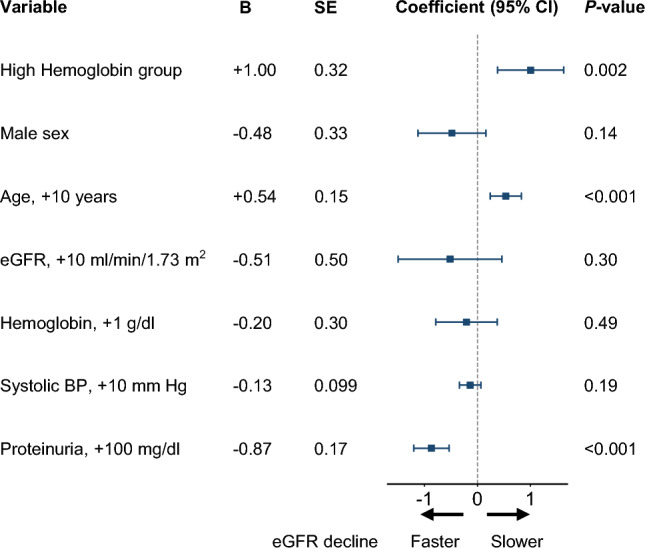
Association between eGFR slope (ml/min/1.73 m2/year) and each covariate in-per protocol set (n = 307). Values are for the interaction term between each variable and time in the mixed-effects model for eGFR. *B* unstandardized regression coefficient, *SE* standard error, *CI* confidence interval, *eGFR* estimated glomerular filtration rate, *BP* blood pressure

The changes in eGFR and proteinuria were also evaluated in the PPS. The crude mean ± SD eGFR slopes in the high- and low-hemoglobin groups were − 2.17 ± 3.05 and − 3.19 ± 2.61 ml/min/1.73 m^2^/year, respectively. In the multivariable analyses, the high-hemoglobin group was associated with improved eGFR slope (coefficient: + 1.00; 95% CI 0.38–1.63; *P* = 0.002), while lower age and higher proteinuria at baseline were associated with faster eGFR decline (Fig. 5). The crude mean ± SD proteinuria slopes in the high- and low-hemoglobin groups were + 0.48 ± 0.080 and + 0.53 ± 0.068 g/gCr/year, respectively, with no significant difference between the study groups in the multivariable model similar to the results in the FAS (Supplementary Table 1). The frequencies of serious adverse events were not significantly different between the two groups (Supplementary Table 2).

## Discussion

In this prespecified secondary analysis of the PREDICT trial, we found that targeting a higher hemoglobin level at 11–13 g/dl with darbepoetin alfa did not improve eGFR and proteinuria slopes as compared to targeting a lower hemoglobin level at 9–11 g/dl in patients with advanced CKD without diabetes. These were in line with the results of the primary analysis in our study that a higher hemoglobin target did not reduce the composite renal outcome [24]. On the other hand, the PPS analysis that excluded patients with off-target hemoglobin levels demonstrated a 36% reduction in the composite renal endpoint and an improvement in eGFR slope of 1 ml/min/1.73 m^2^/year in the high-hemoglobin group compared to the low-hemoglobin group. These were in contrast to the results of previous RCTs indicating that higher hemoglobin targeting was linked to a relatively worse prognosis [11–13, 16].

In the FAS analyses of the eGFR and proteinuria slopes, which was the prespecified secondary endpoints, we could not demonstrate the clear benefit of higher hemoglobin targeting using darbepoetin alfa. The composite renal endpoint was reduced by 22% in the high-hemoglobulin group (vs. low-hemoglobin group) as reported previously [24], and the eGFR decline was 0.59 ml/min/1.73 m^2^/year smaller as presented in this study, although not statistically significant (*P* = 0.08 and *P* = 0.075, respectively). Regarding the proteinuria slope analysis, the mean baseline value was only 58 mg/dL. Therefore, it is difficult to draw any conclusion about the anti-proteinuric effects in this study. Our previous report has also revealed that the rates of CV events were not significantly different between the groups. The PPS analyses in this study suggested that high-hemoglobin targeting was associated with better kidney outcomes among patients with advanced CKD without diabetes and who maintained the target hemoglobin levels without violating the protocol. Darbepoetin alfa, when used properly to maintain the target hemoglobin level at 11–13 g/dl, may exert good effects on the kidney as previously described [14, 15].

As aforementioned, a meta-analysis has demonstrated the potential harm of targeting a higher hemoglobin level (> 13 g/dl) using ESAs in patients with CKD not requiring dialysis [16]. One of the main differences was the target level in the high-hemoglobin group; the target in the three major RCTs was > 13 g/dl [11–13], whereas in our study, the target was not normalization but the middle range at 11–13 g/dl. Taken together with our previous report [24], the PREDICT study demonstrates that maintaining hemoglobin levels at 11–13 g/dl compared with 9–11 g/dl did not at least worsen the prognosis of the patients.

However, the results of the PPS analysis need to be interpreted with caution. Hypo-responsiveness to ESAs was reported to be associated with worse kidney outcomes and CV prognosis [25, 26]. Patients unable to achieve the target hemoglobin level may have been hypo-responsive to ESAs. Especially in the high-hemoglobin group, 57 out of 200 patients were excluded because the lower hemoglobin levels than the target. Therefore, it is still difficult to conclude from the PPS analysis that maintaining hemoglobin levels at 11–13 g/dl protects the kidney more than at 9–11 g/dl.

Concerning the mechanisms of ESAs on the CV prognosis and renal outcomes, both beneficial and harmful effects have been postulated. The beneficial effects for organ protection may be independent of anemia correction but can be attributed to the non-hematological effects of rHuEPO that prevent tissue damage [27]. The potential harm of ESAs may include elevated blood pressure, increased viscosity, and increased platelet number and aggregation [28]. The balance of these factors may have led to different results in RCTs, including this study, depending on the setting of the trial, such as target hemoglobin levels, dosage and kinds of ESA reagents, as well as participants’ characteristics with different risks for CV and renal events. Two RCTs involving patients with CKD receiving kidney transplantation have revealed the renoprotective effects of maintaining normal hemoglobin levels with ESAs on renal outcomes [29, 30]. The participants were relatively young and had a lower risk of CV events than those in other RCTs involving patients with CKD in the pre-dialysis stage.

Regarding the other factors, male sex and higher urinary protein levels at baseline were independently associated with worse renal outcomes in line with those of previous studies [31, 32]. Our study also showed that older age was associated with better renal outcomes after adjusting for other clinical factors. This is probably because those who had hypertensive nephrosclerosis with slower CKD progression were relatively old, while those who had glomerulonephritis with rapid CKD progression were relatively young [33].

Recently, HIF-PHIs have been utilized in treating renal anemia in patients with CKD not requiring dialysis. Many preclinical studies have shown the potential benefit of HIF-PHIs for ischemic organ damage, including the heart and kidney [35–36]. To date, clinical studies have not demonstrated the detrimental effects of HIF-PHIs [17–21, 37, 38]. Since the mechanism of HIF-PHIs function is different from that of ESAs [34, 39, 40], the data of hemoglobin targeting studies obtained by ESA treatment cannot be directly applied to understand the potential benefit or harm of HIF-PHIs in the clinical setting. RCTs to analyze the effects of targeting high vs. low hemoglobin levels on the prognosis of patients with CKD not requiring dialysis using HIF-PHIs will provide clinically useful information. Due to the difference in the relatively lower incidence of CV events in patients with CKD in Japan than in Western countries [41], clinical trials focusing on renal outcomes by targeting high hemoglobin levels with HIF-PHIs should be conducted in each region.

Our study had several potential limitations. First, only patients with CKD in Japan were included in the study, and Japan has a much lower incidence of CV events. Therefore, our results may underestimate the potential harm of CV diseases and may not be generalized to all patients with CKD in other places. Nevertheless, this could also be a strength of our study. Since the rates of CV events in our study were low, only 8% in the high- and 7% in the low-hemoglobin groups [24], the net effects of higher hemoglobin levels on renal outcomes were focused on without considering the indirect effects of CV events. Second, the difference in achieved hemoglobin levels was not more significant than expected, leading to insufficient power to detect the group differences in outcomes. The PREDICT trial was planned and conducted based on the data from the previous study performed in Japan [14], where the difference in the hemoglobin levels between high- and low-hemoglobin groups was approximately 2 g/dl. However, the actual difference in this study was only 1.2 g/dl in the FAS and 1.7 g/dl in the PPS. Third, a prespecified method for making up the PPS may not be the best. Patients who could not reach the higher target levels were excluded due to the violation of protocol. As mentioned above, the patients may have been hyporesponsive to ESA therapy and did not necessarily intend to violate protocols. Due to the selection bias from this, the PPS analysis may overestimate the true effects of targeting higher hemoglobin levels.

## Conclusions

In summary, in this prespecified secondary analysis of the PREDICT trial, the FAS analysis failed to demonstrate that targeting a high hemoglobin level at 11–13 g/dl with darbepoetin alfa reduced the eGFR decline or proteinuria increase as compared to targeting a low hemoglobin level at 9–11 g/dl in patients with advanced CKD without diabetes. In the PPS that excluded patients whose hemoglobin levels did not match the target, the high-hemoglobin group demonstrated better kidney outcomes than the low-hemoglobin group, suggesting the potential benefit of maintaining higher hemoglobin levels. Further prospective studies are needed to determine the optimal hemoglobin target levels using ESAs or HIF-PHIs in patients with CKD not requiring dialysis.

## Supplementary Information

Below is the link to the electronic supplementary material.

Supplementary file1 (DOCX 147 KB)

Supplementary file2 (PDF 61 KB)

Supplementary file3 (PDF 25 KB)
