# Supplementary material for: Higher hemoglobin levels using darbepoetin alfa and kidney outcomes in advanced chronic kidney disease without diabetes: a prespecified secondary analysis of the PREDICT trial
Source: Clin Exp Nephrol. 2023 Jun 8;27(9):757–66. doi: 10.1007/s10157-023-02362-w (PMC10432358; doi:10.1007/s10157-023-02362-w)
Supplement: Supplementary file 1 — Supplementary file1 (DOCX 147 KB) [file 10157_2023_2362_MOESM1_ESM.docx]

**Supplementary Material for:**

**Higher hemoglobin levels using darbepoetin alfa and kidney out outcomes in advanced chronic kidney disease without diabetes:
a prespecified secondary analysis of the PREDICT trial**

**Table of Contents:**

[Supplementary Table 1. Association between proteinuria slope (g/gCr/year) and each covariate in full analysis set and per-protocol set 2](#_Toc132730505)

[Supplementary Table 2. Serious adverse events in per-protocol set 3](#_Toc132730506)

[Supplementary Fig. 1. Darbepoetin alfa dose per visit in per-protocol set (n = 307) 4](#_Toc132730507)

[Supplementary Fig. 2. Association between eGFR slope (ml/min/1.73 m^2^/year) and each covariate in full analysis set (n = 479) 5](#_Toc132730508)

# Supplementary Table 1. Association between proteinuria slope (g/gCr/year) and each covariate in full analysis set and per-protocol set

| **Variable** | **Full analysis set (n = 479)** | | |  | **Per-protocol set (n = 307)** | | |
| --- | --- | --- | --- | --- | --- | --- | --- |
|  | **B** | **SE** | ***P*-value** |  | **B** | **SE** | ***P*-value** |
| High hemoglobin group | 0.032 | 0.15 | 0.83 |  | −0.056 | 0.15 | 0.71 |
| Male sex | 0.21 | 0.15 | 0.17 |  | −0.010 | 0.15 | 0.95 |
| Age, +10 years | 0.074 | 0.070 | 0.29 |  | 0.037 | 0.069 | 0.59 |
| eGFR, +10 ml/min/1.73 m^2^ | −0.48 | 0.23 | 0.042 |  | −0.14 | 0.23 | 0.54 |
| Hemoglobin, +1 g/dl | −0.16 | 0.14 | 0.25 |  | −0.15 | 0.14 | 0.28 |
| Systolic blood pressure, +10 mm Hg | −0.077 | 0.048 | 0.11 |  | −0.055 | 0.049 | 0.26 |
| Proteinuria, +100 mg/dl | 0.024 | 0.075 | 0.74 |  | 0.022 | 0.083 | 0.80 |

Values are for the interaction term between each variable and time in the mixed-effects model for proteinuria.

B, unstandardized regression coefficient; SE, standard error; eGFR estimated glomerular filtration rate.

# Supplementary Table 2. Serious adverse events in per-protocol set

| Adverse events | High hemoglobin group  (n = 136) | Low hemoglobin group  (n = 171) | *P*-value |
| --- | --- | --- | --- |
| Any events | 18 (13%) | 15 (8.8%) | 0.21 |
| Death | 6 (4.4%) | 6 (3.5%) | 0.69 |
| Cardiovascular event | 8 (5.9%) | 8 (4.7%) | 0.64 |
| Thrombosis | 0 (0%) | 0 (0%) | - |
| Pneumonia | 2 (1.5%) | 0 (0%) | 0.11 |
| Malignant neoplasm | 2 (1.5%) | 1 (0.6%) | 0.43 |
| Femur fracture | 1 (0.3%) | 1 (0.6%) | 0.37 |
| Others | 9 (6.6%) | 6 (3.5%) | 0.21 |

Values are n (%). Target hemoglobin levels were 11–13 g/dl in the high-hemoglobin group and 9–11 g/dl in the low-hemoglobin group. *P*-values are for chi-squared test.

# Supplementary Fig. 1. Darbepoetin alfa dose per visit in per-protocol set (n = 307)

The plots with capped spikes are medians with interquartile ranges. Target hemoglobin levels were 11–13 g/dl in the high-hemoglobin group and 9–11 g/dl in the low-hemoglobin group.

# Supplementary Fig. 2. Association between eGFR slope (ml/min/1.73 m^2^/year) and each covariate in full analysis set (n = 479)

Values are for the interaction term between each variable and time in the mixed-effects model for eGFR.

B, unstandardized regression coefficient; SE, standard error; eGFR estimated glomerular.
