# Supplementary figures and images for: Higher hemoglobin levels using darbepoetin alfa and kidney outcomes in advanced chronic kidney disease without diabetes: a prespecified secondary analysis of the PREDICT trial
Source: Clin Exp Nephrol. 2023 Jun 8;27(9):757–66. doi: 10.1007/s10157-023-02362-w (PMC10432358; doi:10.1007/s10157-023-02362-w)

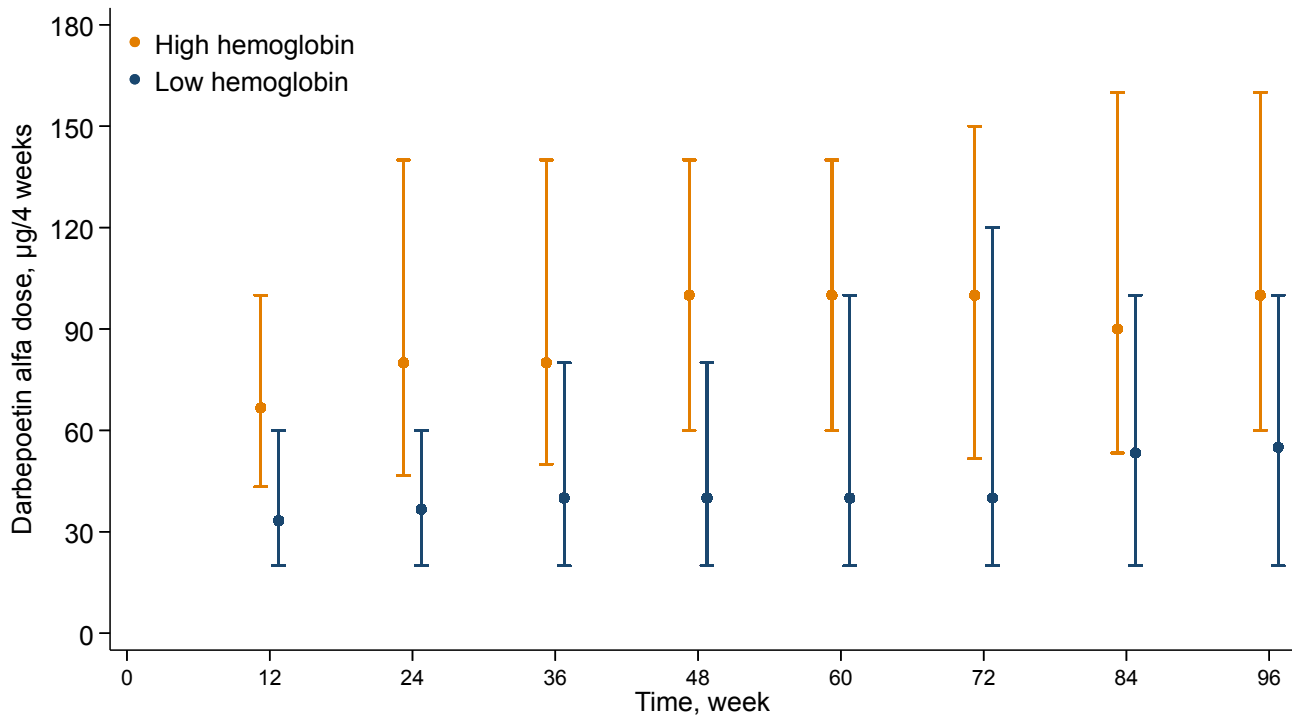

Supplement: Supplementary file 2 — Supplementary file2 (PDF 61 KB) [file 10157_2023_2362_MOESM2_ESM.pdf]

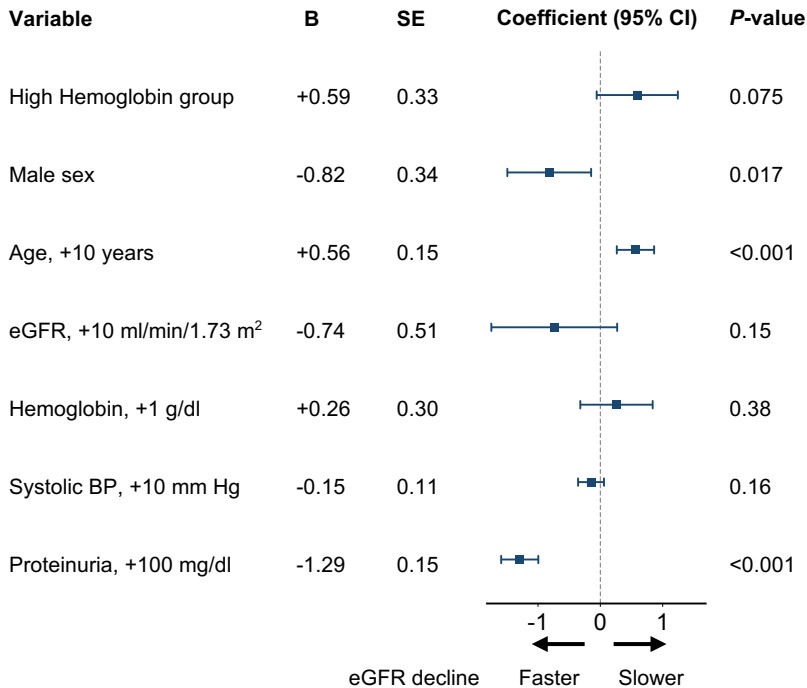

Supplement: Supplementary file 3 — Supplementary file3 (PDF 25 KB) [file 10157_2023_2362_MOESM3_ESM.pdf]
